# Puncture Reduction in Percutaneous Transforaminal Endoscopic Discectomy with HE’s Lumbar LOcation (HELLO) System: A Cadaver Study

**DOI:** 10.1371/journal.pone.0144939

**Published:** 2015-12-16

**Authors:** Guoxin Fan, Xiaofei Guan, Qi Sun, Annan Hu, Yanjie Zhu, Guangfei Gu, Hailong Zhang, Shisheng He

**Affiliations:** Orthopedic Department, Shanghai Tenth People’s Hospital, Tongji University School of Medicine, Shanghai, China; Emory University School of Medicine, UNITED STATES

## Abstract

**Background:**

Percutaneous transforaminal endoscopic discectomy (PTED) usually requires numerous punctures under X-ray fluoroscopy. Repeated puncture will lead to more radiation exposure and reduce the beginners' confidence.

**Objective:**

This cadaver study aimed to investigate the efficacy of HE’s Lumbar Location (HELLO) system in puncture reduction of PTED.

**Study design:**

Cadaver study.

**Setting:**

Comparative groups.

**Methods:**

HELLO system consists of self-made surface locator and puncture locator. One senior surgeon conducted the puncture procedure of PTED on the left side of 20 cadavers at L4/L5 and L5/S1 level with the assistance of HELLO system (Group A). Additionally, the senior surgeon conducted the puncture procedure of PTED on the right side of the cadavers at L4/L5 and L5/S1 level with traditional methods (Group B). On the other hand, an inexperienced surgeon conducted the puncture procedure of PTED on the left side of the cadavers at L4/L5 and L5/S1 level with the assistance of our HELLO system (Group C).

**Results:**

At L4/L5 level, there was significant difference in puncture times between Group A and Group B (P<0.001), but no significant difference was observed between Group A and Group C (P = 0.811). Similarly at L5/S1 level, there was significant difference in puncture times between Group A and Group B (P<0.001), but no significant difference was observed between Group A and Group C (P = 0.981). At L4/L5 level, there was significant difference in fluoroscopy time between Group A and Group B (P<0.001), but no significant difference was observed between Group A and Group C (P = 0.290). Similarly at L5/S1 level, there was significant difference in fluoroscopy time between Group A and Group B (P<0.001), but no significant difference was observed between Group A and Group C (P = 0.523). As for radiation exposure, HELLO system reduced 39%-45% radiation dosage when comparing Group A and Group B, but there was no significant difference in radiation exposure between Group A and Group C whatever at L4/L5 level or L5/S1 level (P>0.05). There was no difference in location time between Group A and Group B or Group A and Group C either at L4/L5 level or L5/S1 level (P>0.05).

**Limitations:**

Small-sample preclinical study.

**Conclusion:**

HELLO system was effective in reducing puncture times, fluoroscopy time and radiation exposure, as well as the difficulty of learning PTED. (2015-RES-127)

## Introduction

For the past decades, with the rapid development of instruments and optic technique, percutaneous endoscopic lumbar discectomy (PELD) has been increasingly applied around the world with the advantages of a small incision, local anesthesia, no neuromuscular retraction, rapid recovery, short operation time and low postoperative expenses[1–4]. Similar to other minimally invasive spinal surgeries, PELD in transforaminal approach (PTED) also requires numerous punctures under X-ray fluoroscopy. Puncture may be repeated for inexperienced surgeons when accurate locating was not achieved, which induces increased injuries of surrounding tissue, more operation time and much more radiation exposure to patients and medical workers.

To improve the accuracy of location and reduce potential radiation exposure, we designed a surface locator and applied it in all kinds of spine surgery, which could accurately locate and mark the target point on the body surface [5–8]. Our previous studies have demonstrated that our surface locator induced less radiation exposure, shorter preoperative time and less frequency for fluoroscopy[7]. However, decreasing the radiation during preoperative location is not enough for PTED, because most repeated fluoroscopy is conducted during the puncture procedure, which usually results in highly cumulative radiation and longer operation time. Therefore, we designed a novel puncture locator combined with surface locator as HE’s Lumbar LOcation (HELLO) system for PTED and performed a preliminary cadaveric study to investigate its efficacy of puncture reduction.

## Materials and Methods

### Specimens

The study was approved by the local Institutional Review Board of Shanghai Tenth People’s Hospital (ethical approval: 2015-RES-127). From July 8^th^ to 26^th^ 2015, all cadavers were donated by the Department of Anatomy, Tongji University School of Medicine and the Second Military Medical University. The Institutional Review Board waived the need for consent from the donors or their kin. All cadaveric specimens had no obvious lumbar vertebra deformity, trauma defects induced by lumbar fracture under fluoroscopy and no previous lumbar surgery. All operating processes and procedures followed the local cadaveric management standards, and the manuscript also followed the reporting guideline (S1 Table).

### HELLO system

**Fig 1:**
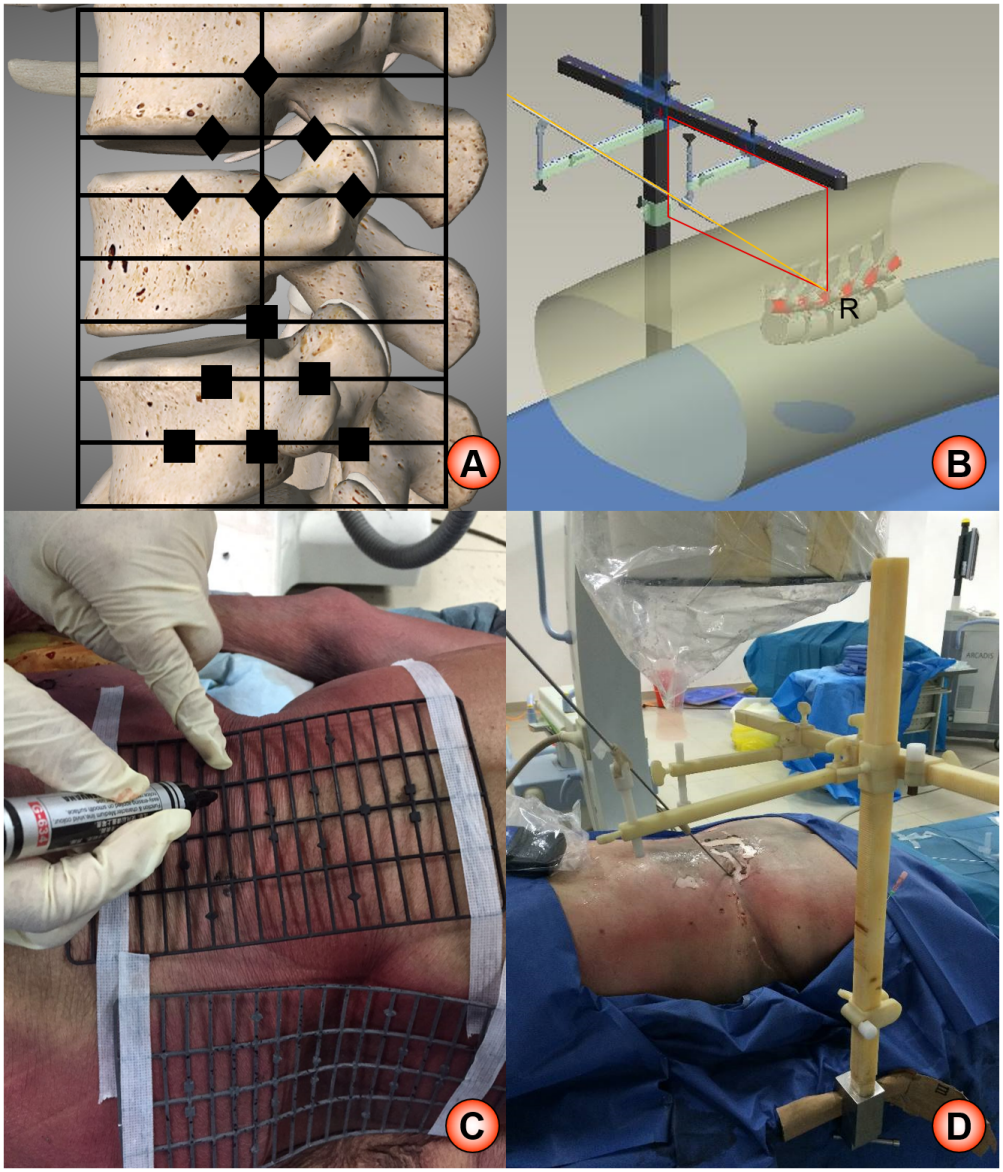
The schematic diagram of HELLO system. A: location theory of surface locator; B: location theory of puncture locator; C: real practice of surface locator; D: real practice of puncture locator.

HELLO system consists of surface locator and puncture locator (Fig 1). Surface locator is made up of radiopaque material, which consists of 19 horizontal rods and 4 longitudinal rods[7]. Each horizontal rod is about 9 cm, whereas each longitudinal rod is about 18 cm. There is about 1-cm gap between each horizontal rod, and different small shape-markers are made on the rods. The stamping die technology and the 1-step forming technology were applied to manufacture the locator. As demonstrated in Fig 1A, the location principle of surface locator is to identify the target with the surrounding rod and shape-markers. The puncture locator is a three-dimensional structure, mainly composed of a vertical beam, a cross beam and two horizontal beams. The location theory of puncture locator is that the target point form a fixed rectangle with the vertical beam and cross beam, and the puncture trajectory go through the target (Fig 1B). The surface locator of HELLO system was used to accurately position the puncture target, and the puncture locator was used to keep the puncture in tract.

**Fig 2:**
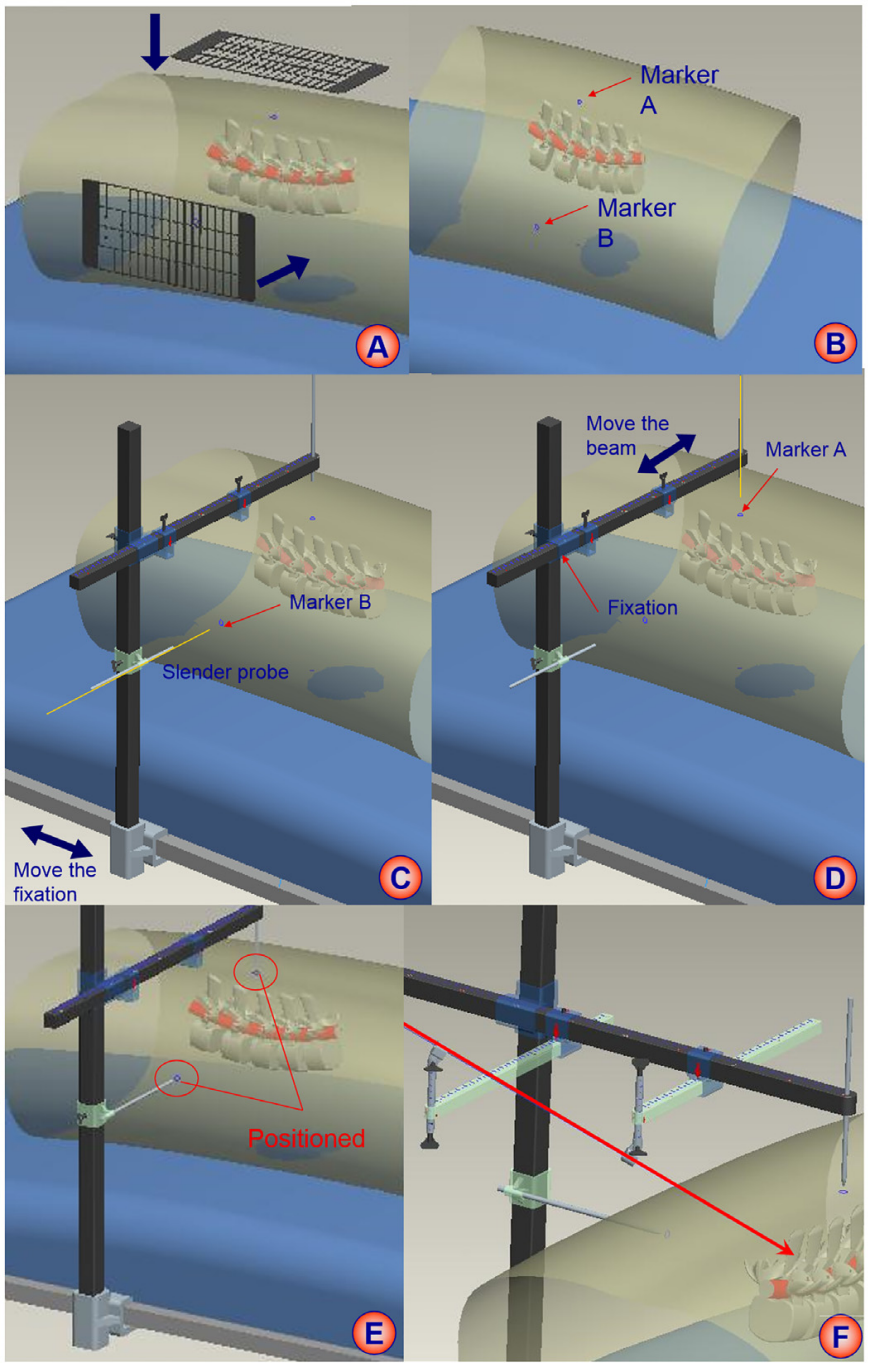
The schematic procedure of HELLO system. A: the attachment of surface locator; B: skin marker of puncture target; C: positioning of puncture locator to the lateral marker; D: positioning of puncture locator to the back marker; E: positioned condition and fixation of puncture locator; F: puncture trajectory to the target.

The procedure of puncture with HELLO system was as follows (Fig 2): Firstly, we used surface locator to determine vertical projection of target point on the cadaveric back under anteroposterior fluoroscopy, and the vertical projection of target point on the lateral cadaveric specimens was also confirmed under lateral fluoroscopy. Then, we marked A and B on the skin of the target projection. Next, we fixed the puncture locator on the horizontal operation table, and vertical beam and cross beam were placed to coincide with A and B. At this time, the target point with the vertical beam and cross beam formed a fixed rectangle, and the two probes and two skin markers were on the long side of the rectangle. Next, the puncture locator in fixed rectangular shape was removed away from the operation table for convenient installation of horizontal beams. According to anatomic structures of puncture segment, the horizontal beams and puncture cannula were adjusted to make two puncture cannulas and target at the same line. Then, we kept the position of horizontal beams and cannula. Finally, locator was fixed on the horizontal operation table, and the vertical beam and cross beam were placed to coincide with A or B. Since the puncture target was accurately located and the puncture could be kept in tract, the appropriate trajectory was finally determined by the entry point. Generally, the distance between the puncture point and the midline of the spinous process was 11–14 centimeters at L4-L5 level, and 12–16 centimeters at L5-S1 level. At that moment, the specific Kirschner was inserted directly to reach target point along the puncture cannula.

### Grouping and puncture procedure

The target puncture segment was L4/L5 and L5/S1 of both sides of each cadaveric specimen. In Group A, the senior surgeon performed the puncture of left L4/L5 and L5/S1 with the assistance of HELLO system. In Group B, the senior surgeon performed the puncture procedure on the right L4/L5 and L5/S1 of cadavers with conventional methods. In Group C, the junior surgeon without PELD experience performed the puncture on the left L4/L5 and L5/S1 of cadavers.

**Fig 3:**
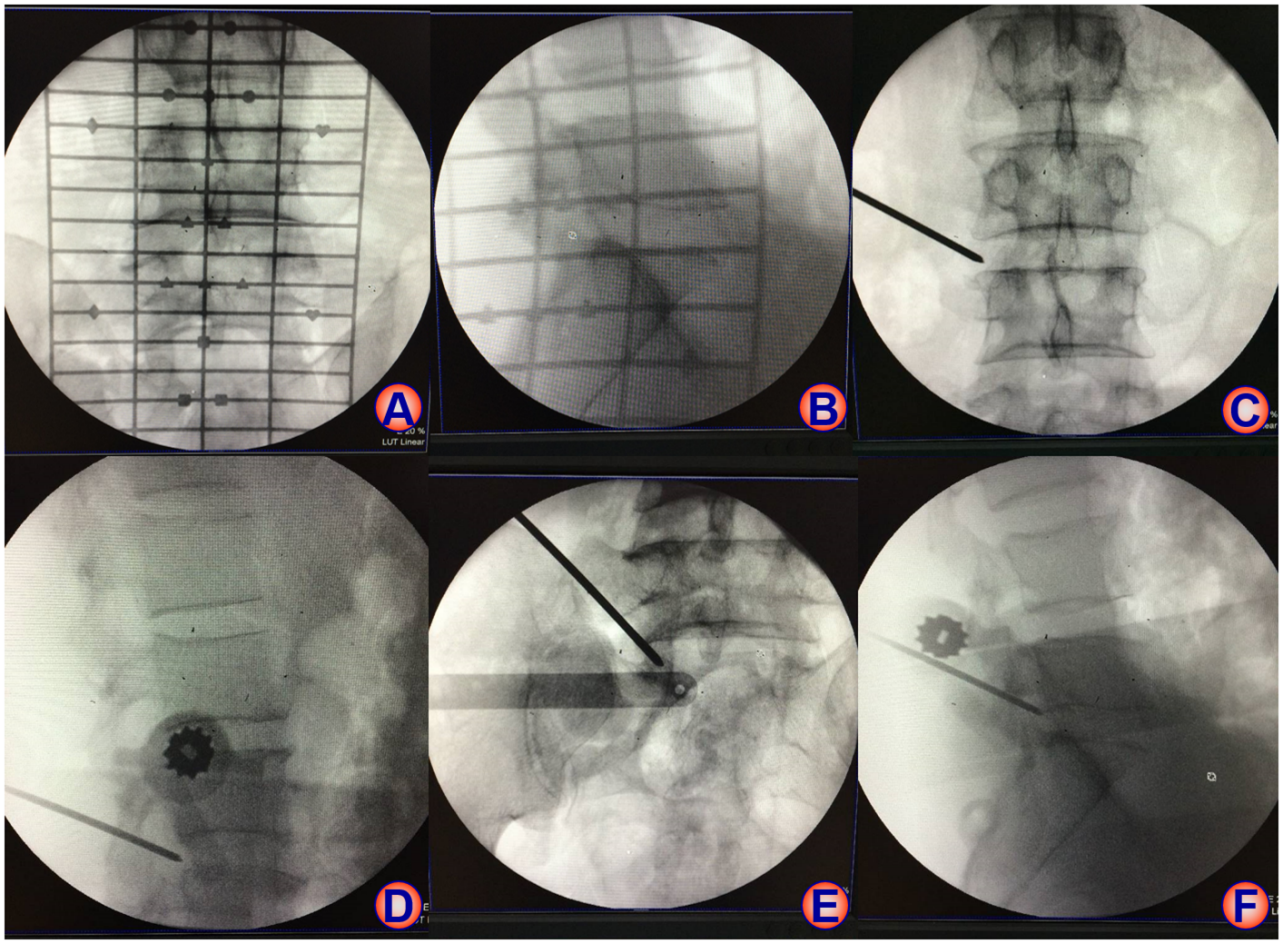
Fluoroscopy of HELLO system on cadavers. A: anteroposterior fluoroscopy of the vertebrae with surface locator; B: lateral fluoroscopy of the vertebrae with surface locator; C: final puncture under anteroposterior fluoroscopy at L4/L5 level; D: final puncture under lateral fluoroscopy at L4/L5 level; E: final puncture under anteroposterior fluoroscopy at L5/S1 level; F: final puncture under lateral fluoroscopy at L5/S1 level.

The cadavers were placed on operation table in prone position, and the C-arm X-ray machine (ARCADIS Varic, Siemens) was used for intraoperative fluoroscopy with fluoroscopy time 1 second each time. The surface locator was used for preoperative location, with which the position of lumbar spinous process, pedicle, intervertebral space, target point and articular process were confirmed and marked (Fig 3A and 3B). Intervertebral foreman and intervertebral space were also marked on the body surface laterally. Group A and Group C underwent locator-assisted puncture by the senior and junior surgeons respectively until kirschner wire was located on the medial pedicle margin in the anteroposterior view and at upper articular process of lower vertebrae on the lateral view (Fig 3C–3F). Group B underwent conventional puncture procedure by senior surgeon with 18G needle inserted into intervertebral foreman of L4/5 and L5/S1 until 18G needle was located on the medial pedicle margin in the anteroposterior view and at upper articular process of lower vertebrae in the lateral view.

### Observational parameters

Puncture times, anteroposterior and lateral fluoroscopy frequency of each segment, the time of locating puncture and accumulated radiation dose were recorded and analyzed. JB4020X-γ personal radiation alarm apparatus (Shanghai Jing Bo Industry & Trade Co., LTD) was used to detect the accumulated radiation dose for each segment.

### Statistical analysis

The software package SPSS 12.0 (USA, SPSS Corporation) was used for statistical analysis. The statistic was demonstrated as Mean±SD. ANOVA test was used to compare the difference among the three groups. P <0.05 was regarded as statistical significance.

## Results

There were 6 cadaveric specimens donated from Tongji University School of Medicine and 14 from the Second Military Medical University. The basic characteristics of included cadavers were demonstrated in Table 1. All three groups completed the puncture procedure at L4/L5 level on 20 cadavers. Only 18 cadavers received L5/S1 punctures, because one had extremely high iliac crest with large transverse process and another had L5 sacralization.

**Table 1:** Basic characteristics of included cadavers.

Variables	Values
Gender	
Male	10
Female	10
Year	52.44±10.05
Condition	
Integrity	9
No upper limbs	2
No lower limbs	5
No extremities	1

In Group A, the fluoroscopy time was 2.70±0.66s for anteroposterior fluoroscopy and 2.75±0.55s for lateral fluoroscopy at L4/L5 level (Table 2). In Group B, the fluoroscopy time was 4.90±1.07s for anteroposterior fluoroscopy and 5.05±1.23s for lateral fluoroscopy at L4/L5 level. In Group C, the fluoroscopy time was 2.90±0.64s for anteroposterior fluoroscopy and 3.05±0.51s for lateral fluoroscopy at L4/L5 level. There was significant difference in fluoroscopy time between Group A and Group B (P = 0.000), but no significant difference was observed in fluoroscopy time between Group B and Group C (P = 0.290). At L5/S1 level, the fluoroscopy time was 3.17±0.71s for anteroposterior fluoroscopy and 3.17±0.71s for lateral fluoroscopy in Group A. In Group B, the fluoroscopy time was 5.56±1.42s for anteroposterior fluoroscopy and 5.61±1.24s for lateral fluoroscopy at L5/S1 level. In Group C, the fluoroscopy time was 3.33±0.77s for anteroposterior fluoroscopy and 3.38±0.85s for lateral fluoroscopy at L5/S1 level. Similarly at L5/S1 level, there was significant difference in fluoroscopy time between Group A and Group B (P = 0.000), but no significant difference was observed in fluoroscopy time between Group B and Group C (P = 0.523).

**Table 2:** Fluoroscopy time of puncture procedure in different groups.

Fluoroscopy time (Mean±SD)	Puncture levels	Anteroposterior fluoroscopy (s)	Lateral fluoroscopy (s)	P value
Group A	L4/L5	2.70±0.66	2.75±0.55	-
Group B		4.90±1.07	5.05±1.23	0.000
Group C		2.90±0.64	3.05±0.51	0.290
Group A	L5/S1	3.17±0.71	3.17±0.71	-
Group B		5.56±1.42	5.61±1.24	0.000
Group C		3.33±0.77	3.38±0.85	0.523

At L4/L5 level, there was significant difference in puncture times between Group A and Group B (P<0.001), but no significant difference was observed between Group A and Group C (P = 0.811) (Table 3). Similarly at L5/S1 level, there was significant difference in puncture times between Group A and Group B (P<0.001), but no significant difference was observed between Group A and Group C (P = 0.981). The location time was 4.39±0.52min in Group A, 4.21±0.65min in Group B, and 4.42±0.38min in Group C at L4/L5 level (Table 4). At L5/S1 level, the location time was 5.26±0.80min in Group A, 4.65±1.19min in Group B, and 5.61±0.77min in Group C. There were no significant differences between Group A and Group B or Group C either at L4/L5 or L5/S1 level (P<0.05). The radiation dosage was 3.48±0.70uSv in Group A, 6.33±1.33uSv in Group B and 3.83±0.58uSv in Group C at L4/L5 level (Table 5). At L5/S1 level, the radiation dosage was 3.99±0.80uSv in Group A, 6.99±1.55uSv in Group B and 4.25±0.89uSv in Group C. In general, HELLO system reduced 42%-45% radiation dosage when comparing Group A and Group B, but there was no significant difference in radiation exposure between Group B and Group C whatever at L4/L5 level or L5/S1 level (P>0.05).

**Table 3:** Puncture times of percutaneous transforaminal endoscopic discectomy in different groups.

Puncture levels	Groups	Puncture time (Mean±SD)	P value
L4/L5	Group A	1.55±0.60	-
	Group B	3.65±1.09	<0.001
	Group C	1.70±0.47	0.811
L5/S1	Group A	2.00±0.59	-
	Group B	4.22±1.22	<0.001
	Group C	2.06±0.73	0.981

**Table 4:** Location time of percutaneous transforaminal endoscopic discectomy in different groups.

Groups	Group A		Group B		Group C	
	L4/L5	L5/S1	L4/L5	L5/S1	L4/L5	L5/S1
Location time (Mean±SD)(min)	4.39±0.52	5.26±0.80	4.21±0.65	4.65±1.19	4.42±0.38	5.61±0.77
P value	-	-	0.283	0.057	0.857	0.277

**Table 5:** Radiation exposure of puncture procedure in different groups.

Groups	Group A		Group B		Group C	
	L4/L5	L5/S1	L4/L5	L5/S1	L4/L5	L5/S1
Radiation dosage (Mean±SD)(uSv)	3.48±0.70	3.99±0.80	6.33±1.33	6.99±1.55	3.83±0.58	4.25±0.89
P value	-	-	0.000	0.000	0.230	0.494

## Discussion

This study demonstrated that HELLO system could significantly reduce puncture times, fluoroscopy time of L4/L5 and L5/S1 and effectively reduce 39%-45% of the radiation dose. Meantime, there was no difference between experienced spine surgeon and inexperienced spine surgeon for puncture with HELLO system whatever in puncture times, fluoroscopy time, radiation dosage or location time, either at L4/L5 level or L5/S1 level.

**Fig 4:**
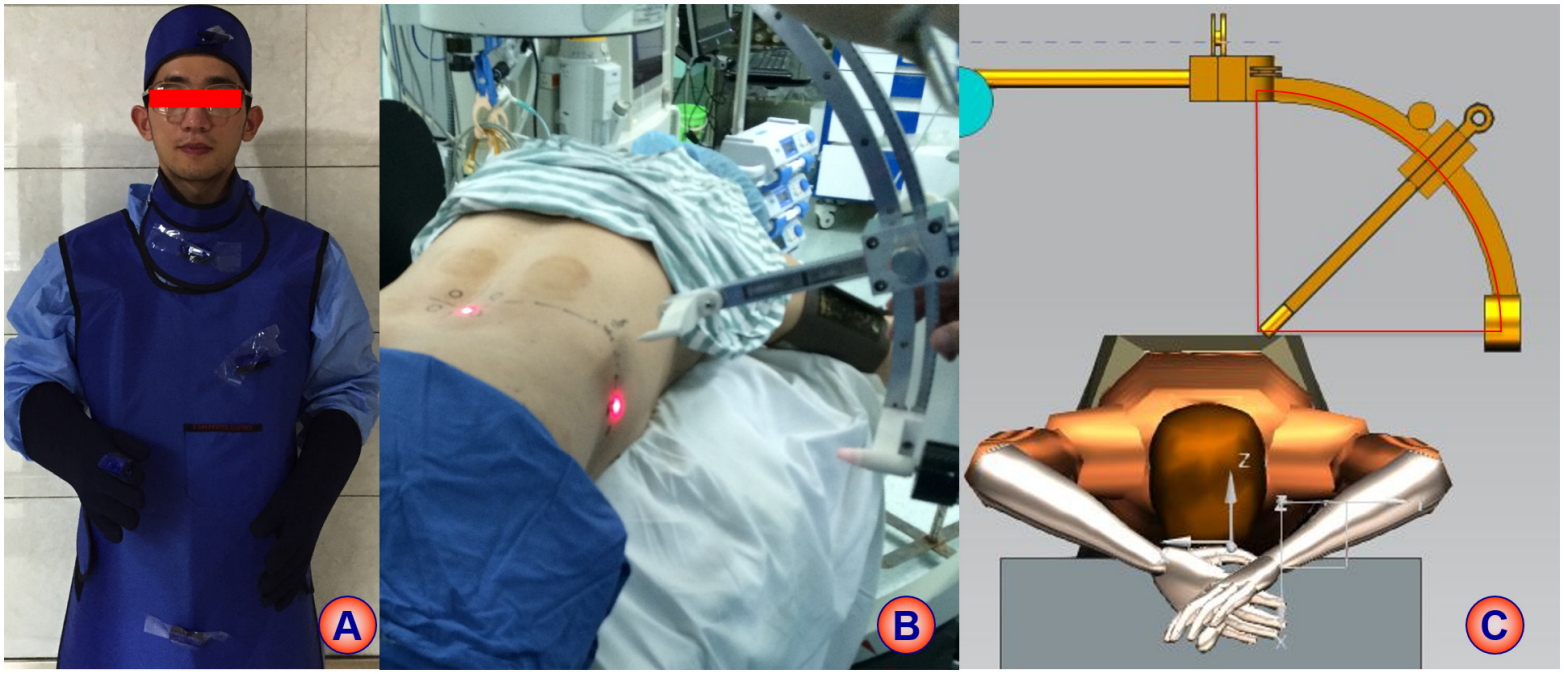
Further registered study with updated puncture locator concerning the radiation exposure on sensitive organs. A: radiation measurement on sensitive organs; B: application of updated puncture locator in clinical practice; C: location theory of second version of puncture locator.

The damage of radiation exposure induced by repeated fluoroscopy in transforaminal endoscopic surgery to patients and surgeons could not be ignored in clinics. The International Commission on Radiological Protection (ICRP) had recommended radiation limits per year for professionals specialized body tissues and organs[9]. Ahn et.al [10] detected the radiation dose for neck, chest, arm and hands of spine surgeons in 30 cases of transforaminal endoscopic surgery, which showed the radiation dose of neck, chest, arm and hands (left and right) were 0.0863 mSv, 0.1890 mSv, 0.0506 mSv, 0.8050 mSv and 0.7363 mSv respectively. The radiation dose of each sensitive organs of spine surgeons in locating puncture procedure was not detected in this study, but overall cumulative radiation dose of the different groups was detected. As the study simulated the puncture part of transforaminal endoscopic surgery, overall cumulative radiation dose of different groups was sufficient to verify the validity of novel puncture locator. We are quantifying the impact of puncture locator on radiation dose of sensitive organs in a registered clinical study (ChiCTR-ICR-15006730) (Fig 4A).

There were various strategies of radiation protection, such as minimizing fluoroscopy frequency and time, keeping away from tube, using low-dose mode and shielding protection[11]. Wearing lead clothes, lead thyroid shield, lead glasses were the most effective methods to reduce the radiation exposure[12]. The surgeon’s position and distance from the tube was considered as the second important method to effectively reduce the radiation exposure. Maintaining three feet away from the tube could greatly reduce the radiation exposure[13]. Different fluoroscopy equipment could lead to different degree of radiation dose[14]. Novel navigation position equipment also reduced the radiation exposure, such as more accuracy and effective O-arm fluoroscopy[15, 16], intraoperative MRI navigation[17] and ultrasonic position technique[10]. However, it must be noted that O-arm fluoroscopy had not been wide applied while intraoperative MRI navigation was also extremely expensive, and ultrasound technology was not well developed. Therefore, HELLO system could be a potential option with the advantages of cheap price, relative portable, reliable practice and well application prospect.

The learning curve for PTED was very steep, because the puncture procedure was very difficult, especially for beginners[18]. Experienced spine surgeons may have a clear understanding of puncture angle required by horizontal beams, and was familiar with the use of C-arm fluoroscopy machine to have a faster switch between lateral fluoroscopy and anteroposterior fluoroscopy. Junior surgeons may perform the puncture procedure more carefully and needs more fluoroscopy on the first 10 cadavers due to lack of PTED experience. Thus, repeated fluoroscopy may lead to more fluoroscopy time, location time and radiation dose. However, our study did not observe significant difference of puncture times, fluoroscopy time, location time and radiation dose between experienced spine surgeons and inexperienced spine surgeons performing puncture with the assistance of HELLO system. In general, HELLO system may reduce the difficulty of PTED for junior surgeons.

When using HELLO system for PTED, the following issues should be noted: 1) The patient should be positioned horizontally in order to improve the accuracy of localization; 2) The image intensifier plane should be paralleled with ground when anteroposterior fluoroscopy was taken; 3) The image intensifier plane should be vertical with ground and paralleled with the long axis of operation table when lateral fluoroscopy was taken. 4) The patients needed to be paralleled with the long axis of operation table to reduce bias induced by surface projection of puncture point; 5) The surface locator need to be fixed on the body surface tightly with adhesive tape. To improve the puncture accuracy and usage convenience, we have updated the design of puncture locator and applied it in clinics (Fig 4B). The second version of puncture locator is based on a fixed 1/4 cyclometer, and the target remains on the sphere center as the puncture trajectory remains on the radius of the cyclometer (Fig 4C). The introduction of HELLO system did not bring additional time to PTED, but it significantly reduced the operation time in our preliminary analysis. This was mainly because HELLO system significantly reduced the puncture times and fluoroscopy.

## Conclusions

HELLO system is effective in reducing puncture times, fluoroscopy time and radiation exposure, as well as the difficulty of learning PTED. A prospective clinical controlled study is ongoing to further confirm the accuracy and efficacy of HELLO system.

## Supporting Information

S1 File This is the supporting information for data statement. (ZIP)

S1 Table This is the reporting guideline. (PDF)
